# Glioma targeting peptide modified apoferritin nanocage

**DOI:** 10.1080/10717544.2018.1464082

**Published:** 2018-05-04

**Authors:** Meifang Zhai, Yuli Wang, Ligang Zhang, Meng Liang, Shiyao Fu, Lin Cui, Meiyan Yang, Wei Gong, Zhiping Li, Lian Yu, Xiangyang Xie, Chunrong Yang, Yang Yang, Chunsheng Gao

**Affiliations:** a College of Pharmacy of Jiamusi University, Jiamusi Heilongjiang, China;; b State Key Laboratory of Toxicology and Medical Countermeasures, Beijing Institute of Pharmacology and Toxicology, Beijing, China;; c Department of Pharmacy, Wuhan General Hospital of the PLA, Wuhan, China;; d Clinical Department, Beijing Huilongguan Hospital, Beijng, China

**Keywords:** Apoferritin nanocage, GKRK peptide, glioma, systemic targeted drug delivery, chemotherapy

## Abstract

Therapeutic outcome for the treatment of glioma was often limited due to the non-targeted nature of drugs and the physiological barriers, including the blood-brain barrier (BBB) and the blood-brain tumor barrier (BBTB). An ideal glioma-targeted delivery system must be sufficiently potent to cross the BBB and BBTB and then target glioma cells with adequate optimized physiochemical properties and biocompatibility. However, it is an enormous challenge to the researchers to engineer the above-mentioned features into a single nanocarrier particle. New frontiers in nanomedicine are advancing the research of new biomaterials. In this study, we demonstrate a strategy for glioma targeting by encapsulating vincristine sulfate (VCR) into a naturally available apoferritin nanocage-based drug delivery system with the modification of GKRK peptide ligand (GKRK-APO). Apoferritin (APO), an endogenous nanosize spherical protein, can specifically bind to brain endothelial cells and glioma cells via interacting with the transferrin receptor 1 (TfR1). GKRK is a peptide ligand of heparan sulfate proteoglycan (HSPG) over-expressed on angiogenesis and glioma, presenting excellent glioma-homing property. By combining the dual-targeting delivery effect of GKRK peptide and parent APO, GKRK-APO displayed higher glioma localization than that of parent APO. After loading with VCR, GKRK-APO showed the most favorable antiglioma effect *in vitro* and *in vivo.* These results demonstrated that GKRK-APO is an important potential drug delivery system for glioma-targeted therapy.

## Introduction

The efficient treatment for glioma, which is the most frequent primary malignant brain tumor with a median survival less than 2 years after first diagnosis, remains a challenging task. Glioma is difficult to treat because of the special pathological and physiological characteristics. Due to the infiltrate growth of glioma, it is hard to completely remove the glioma through surgery (Donahue et al., 2008). Chemotherapy is indispensable for subsequent treatment after surgery. Unfortunately, the clinical therapeutic effect of glioma by chemotherapy is very unsatisfying. The blood-brain barrier (BBB) rigorously prevents 98% of drugs from reaching the infiltrative glioma cells, resulting in glioma recurrence after surgical resection. Moreover, the non-targeted nature of drugs often causes severe side effects because they produce a similar cytotoxicity in both cancerous and healthy cell.

To meet this challenge, many attempts have focused on nanocarriers (Kreuter, 2001). Over the past few decades, various types of nanocarriers for glioma therapy such as liposomes, mesoporous silica nanoparticles and polymeric particles have been fabricated from various materials. These nanocarriers has attracted considerable attention and been developed to bridge the requirements of glioma treatments, such as improving the transportation of drugs across the BBB or selectively accumulating at glioma site. However, issues related to their synthetic materials can limit their effects and cause toxicology issues (Nativo et al., 2008; Guerrero-Cázares et al., 2014).

To circumvent the obstacles imposed by nanocarriers that consist of synthetic materials, suitable nanocarriers for efficient drug delivery should be developed using natural materials, which are considered relatively safe for the organism (Moghimi et al., 2005; Symonds et al., 2005; Gao et al., 2011). In recent years, many endogenous transporters that facilitate the uptake of nutrients and minerals have been revealed in the cerebral endothelium (Wong et al., 2012). Among these endogenous transporters, ferritin has attracted tremendous attention for its application in payload loading and delivery owing to its unique features (He & Marles-Wright, 2015). (1) Biosafety: Ferritin is a major iron storage protein found in many living organisms, including human beings that would not activate inflammatory or immunological responses (De Groot & Scott, 2007). (2) Nanostructure: When expressed artificially in iron-free conditions, the resultant apoferritin is hollow. This hollow architecture provides two interfaces, one outside and one inside, for possible functional modification and loading (Zhen et al., 2013). (3) Size: Ferritin is small (outer diameter of 12 nm), spherical, and highly homogeneous (Joyce, 2005). This may lead to a longer circulation half-life and, eventually, to better accumulation rates, compared with synthetic nanocarriers. (4) Targeting: It was recently reported that ferritin can bind to cells via interacting with the transferrin receptor 1 (TfR1) (Li et al., 2010). Because of the high expression of TfR1 in brain endothelial cells, apoferritin can also cross the BBB with TfR1 and deliver iron to the brain (Burdo & Connor, 2003). Therefore, the natural cellular uptake of parent apoferritin provides a biological pathway to facilitate brain-targeted delivery of cargo loaded into the cavity through TfR1-mediated endocytosis. The properties of parent apoferritin as mentioned above have been taken as design cues to devise the next-generation targeted delivery platforms (Tosi et al., 2016). The delivery potential of parent apoferritin has been explored and many payload, such as radioisotopes (Hainfeld, 1992), antibiotics (Lown, 1993), alkylating agents (Siddik, 2002), and siRNA (Li et al., 2016), can be loaded into the apoferritin nanocage for tumor-targeted diagnosis and therapy.

However, it is now increasingly acknowledged that glioma is genetically heterogeneous and complex, and shows variations in the expression of the bookmakers postulated to be important for active targeting strategy. For example, the dysregulated TfR expression in glioma cells is often only 3–5-fold higher than that in normal cells (Pang et al., 2011). This inadequate expression of the receptors on glioma tissue usually compromised the efficacy of mono-targeting glioma drug delivery. In addition, the special microenvironment in the brain leads to relatively narrower fenestrae in glioma neovasculature than those in peripheral tumors and thus forms the blood-brain tumor barrier (BBTB), impeding the penetration of nanocarriers into glioma cells. So far, the use of parent apoferritin as a carrier for glioma-targeted delivery has not previously been tested *in vivo*. To augment the antiglioma therapy efficacy, it is essential to develop a dual-targeting glioma drug delivery system with improved glioma delivery capablitiy. GKRK peptide, which was discovered by phage display (Hoffman et al., 2003), could bind to heparan sulfate proteoglycan (HSPG) with high affinities and specificities. Since HSPG is over-expressed in glioma cells and angiogenesis (Jarvinen & Ruoslahti, 2007; Zhang et al., 2014), GKRK peptide modified nanocarriers could efficiently inhibit glioma growth by crossing the BBTB and targeting glioma cells and angiogenesis (Hu et al., 2013). Inspired by these results, a rational strategy was employed to take advantage of the parent apoferritin nanocage (APO) with GKRK peptide functionalization to enhance glioma therapy in our research. Parent apoferritin carries reactive groups (e.g. sulfhydryl and amino groups) on its outside surfaces that can be used for ligand binding by covalent linkage. GKRK peptide was functionalized to apoferritin via a sulfhydryl-maleimide coupling reaction aiming at obtaining precise dual-targeting efficacy. The GKRK peptide modified APO (abbreviated as GKRK-APO) may efficiently overcome multiple barriers (BBB and BBTB) and target glioma cells via interacting with the TfR1 and HSPG. This dual-targeting design is intended to improve the selective delivery to the glioma site, and to reduce intrinsic toxicity to healthy cells beyond the reliance upon the EPR effect and mono-targeting modification. Using bEnd.3 cells (over-expressed TfR1) as the model of brain endothelial cells, HUVEC cells (over-expressed HSPG) as the model of neovascular endothelial cells, and U87MG cells (over-expressed HSPG and TfR1) as the model of glioma cells model, cellular association and internalization mechanism was investigated bEnd.3 cells, HUVEC cells and U87MG cells by using Cy5.5 as the fluorescence probe. *In vivo* bio-distribution of GKRK-APO was also studied by taking Cy5.5 as near-infrared probe. After encapsulating vincristine sulfate (VCR) as the model drug, the anti-tumor efficacy of GKRK-APO was evaluated on *in vivo* intracranial U87MG tumor-bearing mice. Herein, we report the first study on functional apoferritin nanocage as a glioma-targeted delivery system. This study will help elucidate the functions of apoferritin nanocage and allow for its application as a powerful nanoplatform for brain tumor diagnosis and therapy.

## Experimental materials

### Materials

Apoferritin from equine spleen (horse spleen apoferritin) 0.2 μm filtered and all chemicals were obtained from Sigma-Aldrich (St. Louis, MO), unless otherwise stated. Sulfate vincristine (VCR) was obtained from Baiyunshan Co. (Guangzhou, China). Maleimided GKRK peptide (GKRK-MAL) was synthesized by Cybertron Medical Technology Co. (Beijing, China). All chemicals were of reagent grade and were obtained from Sigma-Aldrich, unless otherwise stated.

HUVEC cells, U87MG, and bEnd.3 cells were provided by the Cell Resource Centre of IBMS (Beijing, China) and cultured in Dulbecco’s modified Eagle’s medium (DMEM) containing 10% FBS (Gibco) and 1% antibiotics at 37 °C in humidified atmosphere and 5% CO_2_.

Female Sprague–Dawley (SD) rats (180–220 g) and Female BALB/c nude mice (weighing 18–22 g) were purchased from Vital River Laboratories (Beijing, China). All procedures involving animal housing and treatment were approved by the Animal Care and Use Ethics Committee of the Academy of Military Medical Sciences.

## Methods

### Preparation of GKRK-APO

GKRK-APO was prepared by coupling GKRK-MAL to APO through sulfhydryl-maleimide coupling reaction (Zhen et al., 2013). Briefly, GKRK-MAL was conjugated with APO (molar ratio, 0.5%, 1%, 3%, and 6%) in phosphate buffer (pH 8.0) at 4 °C overnight while stirring. The reaction solution was dialyzed (molecular weight cut-off 3.5 kDa, Thermo Scientific, Grand Island, NY) in distilled water for 24 h to remove the excess peptides.

### Labeling of nanocarrier

To monitor the *in vitro* and *in vivo* behaviors, GKRK-APO or APO was labeled with the fluorescent dye Cy5.5 through NHS-amino coupling reaction. Briefly, the Cy5.5-NHS (Lumiprobe, Hunt Valley, MD) was dissolved in dry DMSO and added to GKRK-APO or APO solution (PBS, pH 8.0), at a dye to GKRK-APO molar ratio of 10:1. The mixture was gently stirred overnight at 4 °C in the dark and then dialyzed in a dialysis bag to remove free dyes.

### Identification of peptide modifications

Purified HSPG were immobilized in the wells of microtiter plates (2 mg/mL, 50 mL/well) by adsorption overnight before blocking with casein blocker (Pierce, St.Louis, MO). Cy5.5-labeled GKRK-APO were re-suspended in binding buffer (50 mM Tris, pH 8.0, 150 mM NaCl, 1 mM MgCl_2_, 1 mM CaCl_2_, 0.5 mM MnCl_2_) and added to the microtiter wells (Kessler et al., 2005). After incubation for 2 h, the unbound GKRK-APO was removed by washing the wells with the binding buffer twice. Bound GKRK-APO was then quantitated by determining the fluorescence intensity. To detect the binding specificity of GKRK-APO to HSPG, the HSPG-coated wells were pre-incubated with GKRK peptide which shows competitive binding to HSPG.

### Loading drug

The loading of VCR into the cavity of GKRK-APO and APO was prepared as described by previous report (Kilic et al., 2011), with minor modifications. Apoferritin from equine spleen (horse spleen apoferritin) was obtained from Sigma-Aldrich. Briefly, 100 μL of APO (10 mg) was dissolved in 0.15 M NaCl. Then, VCR was added into the solution at a final concentration of 1 mg/mL and stirred for 30 min. Subsequently, the pH of the mixed solution was adjusted to 2.0 using 0.1 M HCl and stirred for 10 min. In order to reassemble the protein to its native nanosphere form, the pH of the solution was then slowly increased to 7.4 by adding 0.1 M NaOH solution under constant stirring and continuous monitoring of pH through pH meter. The excessive VCR and the drug molecules outside of the protein shell were removed by dialysis against 0.9% NaCl solution. Finally, the VCR-loaded GKRK-APO and VCR-loaded APO were filtration sterilized by 0.2 μm filter and subpackaged to aseptic vials.

### *In vitro* of VCR release

Dialysis was performed to investigate the *in vitro* release of VCR from the VCR-loaded GKRK-APO or APO. The release medium was PBS buffer (0.1 M, pH 7.4) or acetate buffer (0.1 M, pH 5.0), respectively. About 0.5 mL of VCR-loaded GKRK-APO or VCR-loaded APO was added to dialysis bag with molecular weight cut-off 12,000–14,000. The dialysis bag was then placed in a flask filled with 30 mL medium at 37 °C. At predetermined intervals, 800 μL of medium was drawn out and replenished with the same volume of fresh medium. The released free VCR at different incubation times was assayed by HPLC, as previously reported (Li et al., 2016).

### Characterization of GKRK-APO

The mean diameter and particle distribution of these GKRK-APO carriers were measured by dynamic light scattering (Nanophox, Sympatec GmbH, Germany). Morphology of the VCR-loaded GKRK-APO was characterized via an atomic force microscopy (AFM) (NanoWizarc, JPK Ltd., Berlin, Germany) and transmission electron microscopy (TEM) (HITACHI, H-7650, Tokyo, Japan), respectively. The stability of VCR-loaded GKRK-APO in 10% FBS was evaluated using a Turbiscan Lab^®^ Expert (Formulaction, L'Union, France). The analysis of stability was carried out by the software of the instrument, as a variation of back-scattering (Δ*BS*) profiles.

The VCR encapsulation efficiency (EE) of VCR-loaded GKRK-APO and VCR-loaded APO was calculated using the following equation:

$$EE\%=(W_{total\;drug}-W_{free\;drug})/W_{total\;drug}\times 100\%$$

where, *W*_total drug_ and *W*_free drug_ represent the total drug in APO and the amount of free drug in the ultrafiltrate, respectively.

### Effect of peptide density on cellular uptake of APO

To investigate the effect of GKRK peptide density on cellular uptake, Cy5.5-labeled GKRK-APO was prepared at different peptide densities (0.5%, 1%, 2%, 3%, and 4%, molar ratio). U87MG cells were seeded into 12-well plates for flow cytometry (FCM) (BD FACSCalibur, San Jose, CA). The concentration of Cy5.5 was 150 ng/mL.

### Binding of nanocarrier to cells

In order to assess the binding affinity of GKRK-APO or APO to cells, different 5 μM Cy5.5-labeled GKRK-APO or Cy5.5-labeled APO were incubated with three types of cells (U87MG cells, HUVEC cells, and bEnd.3 cells) at 37 °C for 2 h, respectively. The cells were washed three times with cold PBS, then centrifuged and re-suspended with PBS for qualitative analysis by confocal laser scanning microscopy (CLMS) (UltraVIEW Vox, PerkinElmer,  ‎Waltham, MA) and quantitative analysis by FCM.

### Cytotoxicity

HUVEC cells or U87 MG cells were seeded into 96-well plates at the density of 5000 cells/well and incubated for 24 h at 37 °C. Then cells were treated with various samples (blank APO, blank GKRK-APO, free VCR, VCR-loaded APO, and VCR-loaded GKRK-APO) at a range of concentrations. The cytotoxicity of each sample was determined by MTT method in triplicate.

### Transport across the *in vitro* BBB and BBTB models

To establish *in vitro* BBB model, a bEnd.3/U87MG co-culture BBB model was established according to previous reports (Li et al., 2014). Briefly, bEnd.3 cells were seeded on the upper side at 1.0 × 10^5^ cells per insert (Corning, NY). U87MG cells were seeded on the basolateral compartment of the insert at 2000 cells/compartment. The cell monolayer integrity was measured by transendothelial electrical resistance (TEER). BBB model was considered constructed successfully when the TEER value reached 200 Ω·cm^2^. To establish *in vitro* BBTB model, U87MG cells were plated onto the lower chamber, and HUVEC cells were seeded into the upper inserts of transwell with a density of 1:5 HUVEC/U87MG cells ratio (Khodarev et al., 2003). After incubation for 5 days, these models were used for experiments (TEERs of the BBB and BBTB models were 206.4 ± 15.1 and 173.8 ± 11.7 Ω × cm^2^, respectively). To confirm that TfR1 and HSPG were expressed in both models, several U87MG cell monolayers in both models were removed, and the expression of TfR1 and HSPG in each model was measured by TfR1 ELISA kit (Shanghai Jingkang Biologic Company, Shanghai, China) and HSPG Human ELISA kit (Wuhan Cloud-Clone Corporation, Wuhan, China), respectively. The measuring processes were performed according to the manufacturer’s instructions. The TfR1 concentration in BBB and BTB models was 43 ± 5 and 46 ± 5 ng/mL, respectively. The HSPG concentration in BBB and BTB models was 210 ± 15 and 220 ± 18 pg/mL, respectively. These results confirmed that TfR1 and HSPG were highly expressed in both models.

The culture medium in the upper chambers was changed by 50 μM Cy5.5-labeled GKRK-APO or Cy5.5-labeled APO in 10% FBS containing DMEM. At definite time points, the U87MG cells on the bottom of the cell were immediately visualized by CLSM. To assess the antiproliferative effect of free VCR, VCR-loaded GKRK-APO, and VCR-loaded APO against U87MG cells after penetrating the BBB model or BBTB model, a sulforhodamine-B staining assay was applied. The various samples (free VCR, VCR-loaded GKRK-APO, and VCR-loaded APO) were added to the apical compartment of the BBB model or BBTB model, respectively. The final concentration of VCR was 250 ng/mL. After 48 h, the percentage of surviving glioma U87MG cells in the basolateral compartment was determined by the sulforhodamine-B staining assay (Li et al., 2014).

### Pharmacokinetic studies

The pharmacokinetic profiles of VCR-loaded nanoparticles were measured in SD rats with a single dose of 1 mg/kg VCR (i.v. via tail vein, diluted to 0.5 mL by physiological saline). Blood was sampled from the retro-orbital sinus at different time points (0.08, 0.25, 0.5, 1, 2, 3, 4, 6, 8, 12, 24 h). To prepare samples for analysis, 0.1 mL the internal standard (I.S.) solution (vinblastine sulfate, 40 ng/mL) was added to 100 µL plasma sample in a 1.5 mL test tube. The sample mixture was deproteinized with 0.8 mL of methanol and vortex-mixed for approximately 1 min, and the precipitate was removed by centrifugation at 12,000 rpm (revolutions per minute) for 10 min. Then 800 µL of supernatant was transferred to another clean test tube and evaporated to dryness at 37 °C with a CentriVap Concentrator (Labconco Corporation, Kansas City, MO). The dry residue was reconstituted in 100 µL of the mobile phase, vortex-mixed, and centrifuged at 12,000 rpm for another 10 min. Twenty microliters of the clean supernatant was injected into the LC/MS/MS for analysis as described previously (Guilhaumou et al., 2010).

### Tissue biodistribution

The intracranial glioma-bearing mice model was established as described previously (Xin et al., 2011). The mice were randomly divided into three groups, intravenously administrated with free VCR, VCR-loaded APO, and VCR-loaded GKRK-APO at a dose of 1 mg/kg VCR (diluted to 0.2 mL by physiological saline), respectively. Thirty minutes after the i.v. injection, three mice of each group were sacrificed with the representative organs collected and stored at –20 °C until analysis. VCR was extracted from the mouse tissues by protein precipitation (Ernsting et al., 2012). The contents of VCR were analyzed by a LC-MS/MS as described previously (Guilhaumou et al., 2010).

### Live animal imaging

The intracranial glioma-bearing mice were administered Cy5.5-labeled GKRK-APO or Cy5.5-labeled APO (diluted to 0.2 mL by physiological saline) via tail vein injection. Thirty minutes after the i.v. injection, *in vivo* imaging was performed with an IVIS^®^ Spectrum-CT. Fluorescent signals were quantified using Living Image^®^ software (Caliper, Alameda, CA).

### Glioma distribution

The Cy5.5-labeled GKRK-APO or Cy5.5-labeled APO (0.5 mg/kg, diluted to 0.1 mL by physiological saline) was administered via tail vein injection to the mice. After 30 min, the intracranial glioma-bearing mice were anesthetized, and the hearts were perfused with saline, which was followed by 4% paraformaldehyde. The brains were removed for consecutively preparing 5-μm-thick frozen sections. Nuclei were stained with DAPI. The distribution of fluorescence was observed using CLSM.

### *In vivo* antiglioma effect

The intracranial glioma-bearing mice were randomly divided into the following four groups (10 mice per group): Free VCR gourp, VCR-loaded GKRK-APO group, and VCR-loaded APO group and physiological saline group. Eight days after cell injections, each mouse received a dose of 1 mg/kg four times every 2 days. At day 16, four mice from each group were anesthetized and brain cancer was assessed by magnetic resonance imaging (MRI) (Siemens, Munich, Germany) with measurement of the tumor diameter. Glioma inhibition was calculated using the formula: *R*_v_= (*V*_drug_/*V*_saline_) × 100%. Where *V*_drug_ is the glioma volume after treatment with drug and *V*_saline_ is the glioma volume after treatment with physiological saline. The remaining six mice in each group were used to monitor survival. The survival time was calculated from day 0 (tumor inoculation) to the day of death. Kaplan–Meier survival curves were plotted for each group. Meanwhile, the body weight of each mouse was measured daily.

### Histology of brain tumors

After the treatments were finished, the mice were sacrificed to collect the brains. Brain tumors were fixed in 10% buffered formalin, embedded in paraffin, and sectioned at 5 μm thickness. Sections were stained with Hematoxylin and Eosin (HE). The tumor histology was viewed and imaged under optical microscopy (Olympus Company, Tokyo, Japan).

### Apoptosis assay (TUNEL)

Apoptotic cells in glioma were detected via TUNEL assays using an *in situ* cell death detection kit following the manufacturer’s instructions prior to observation under CLSM.

### Safety assay

In order to study the toxicity of VCR-loaded GKRK-APO, the mice were administered with free VCR, VCR-loaded GKRK-APO, and VCR-loaded APO at a single VCR dose of 1 mg/kg body weight via the tail vein, respectively. The mice treated with PBS were investigated as a control group. After mice were sacrificed, samples of brain, heart, liver, kidney, and lung tissue were collected and routinely stained with hematoxylin and eosin (H&E).

### Statistical analysis

Data were expressed as the means ± standard deviation (SD). The *C*_max_ and AUC_(0-_*_t_*_)_ were calculated by Data Analysis System 3.16 (DAS 3.16, Clinical drug research center, Shanghai University of Traditional Chinese Medicine, Shanghai, China). A one-way ANOVA was used to analyze the differences among groups. The significance was set at *p* = .05.

## Results and discussion

### Identification and characterization of GKRK-APO

**Figure 1.**
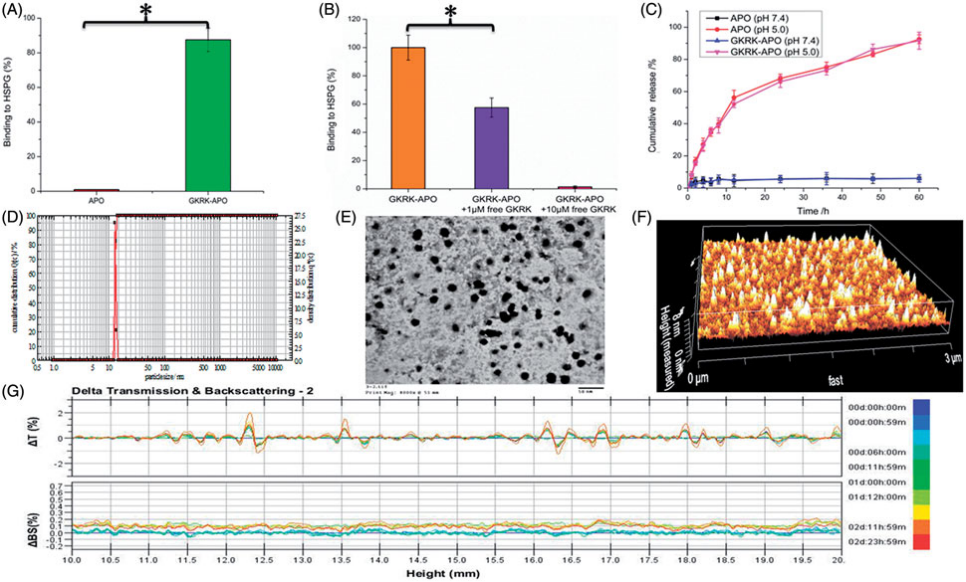
Physicochemical characterization of GKRK-APO. Binding of Cy5.5-labeled APO or Cy5.5-labeled GKRK-APO to immobilized HSPG integrin was assessed by measuring the fluorescence of the wells following washing to remove unbound samples (A). Binding of Cy5.5-labeled GKRK-APO (2 μM) to immobilized HSPG was significantly inhibited by the free GKRK peptide in a dose-dependent manner (B). In vitro release of VCR from GKRK-APO and APO at pH 5.0 and pH 7.0 at 37 °C, respectively (C). Particle size distribution of VCRloaded GKRK-APO (D). Morphological appearance of VCR-loaded GKRK-APO based on TEM (E) and AFM (F). Stability of VCR-loaded GKRK-APO in the presence of 10% FBS. The transmission and backscattering profiles were measured at each time point using a Turbiscan Lab^®^ Expert analyser (G). The data are presented as the means ± SD (n = 3). * indicates P< 0.05.

Because of the high activity of sulfhydryl group (-SH) in APO, the surface of APO can be easily used for ligand binding by sulfhydryl-maleimide coupling reaction. The GKRK peptide was introduced maleimide group (-MAL), and the peptide was conjugated to APO via the sulfhydryl-maleimide coupling reaction, which enabled GKRK peptide to be conjugated at a specific site (-MAL). To confirm GKRK peptide was functionalized to APO, we analyzed the binding ability of the GKRK-APO to purified HSPG. The GKRK-APO were labeled with the fluorescent agent Cy5.5 and incubated with immobilized HSPG in the wells of a microtiter plate for 2 h. The GKRK-APO strongly bound to the HSPG-coated well (Figure 1(A)), but no detectable binding was observed in the APO group. The binding of GKRK-APO to HSPG was blocked by free GKRK peptide in a dose-dependent manner (Figure 1(B)), consistent with the modification of GKRK on the APO surface.

The physico-chemical properties of the two distinct apoferritin nanocage formulations are summarized in Table 1. The subunits of apoferritin nanocage can be disassembled at strong acidic environments (pH 2.0) and reassembled by returning the pH to physiological conditions (pH 7.4) in a shape-memory fashion. Therefore, the payload encapsulation/release in/from apoferritin nanocage depends on the pH (Kilic et al., 2011). In this study, we used the pH method for VCR loading into GKRK-APO and APO. The VCR encapsulation efficiency (EE) of GKRK-APO and APO was approximately 39.8 ± 0.9% and 40.3 ± 1.2%, respectively. The modifications of GKRK on the surfaces of the APO did not affect the ultimate encapsulation efficiency. After entering the intracellular region, VCR-loaded GKRK-APO or VCR-loaded APO is engulfed by lysosomes. Lysosomal acidification significantly contributes to the disassembly of GKRK-APO or APO, and release of VCR. To verify the pH-dependent drug release of VCR-loaded GKRK-APO, the PBS buffer (0.1 M, pH 7.4) and acetate buffer (0.1 M, pH 5.0) were employed at 37 °C to mimic the physiological conditions and lysosomal situation, respectively (Kilic et al., 2011). As seen in Figure 1(C), at pH 7.4, no significant release of free VCR was observed over 60 h of incubation. Delayed drug release would be beneficial for the prevention of rapid leakage during the process of drug delivery through the circulatory system and for the accumulation of a drug in the cancer tissue. In contrast, at pH 5.0, free VCR was detected and reached a maximum release of 91.60 ± 5.28% for GKRK-APO and 92.42 ± 4.79% for APO at 60 h, respectively. These results suggest that a potential lysosome-based drug release mechanism exists for the GKRK-APO drug delivery vehicle. In addition, there were no pronounced differences in VCR release behavior between GKRK-APO and APO at each time point (*p* > .05), which showed that the conjugation of GKRK peptide to APO did not affect drug releasing.

**Table 1.** Characteristics of the nanocarriers.

Sample ID	Diameter (nm)	Polydispersity index	Encapsulation efficiency (%)
APO	12.58 ± 0.11	0.08 ± 0.01	40.4 ± 1.1
GKRK-APO	12.77 ± 0.14	0.10 ± 0.01	39.8 ± 0.9

The data are expressed as the mean ± SD for three different preparations (*n* = 3).

For the nanocarriers, the particle size of nanoparticle is a precondition and crucial factor that determines the fate of nanocarriers *in vivo* and *in vitro*. After an *in vitro* releasing study, the particle size of GKRK-APO was further analyzed by a laser particle analyzer. The mean particle size of the GKRK-APO was 12.77 ± 0.14 nm, and it had a narrow size distribution (the polydispersity index was 0.10 ± 0.01) (Figure 1(D)). This particle size was suitable for delivery in the circulation because this size was sufficiently small to cross into tissues, approach cell surface receptors, and facilitate intracellular transport (Zhao et al., 2012). TEM (Figure 1(E)) and AFM (Figure 1(F)) observation confirmed the results of laser particle analyzer. The GKRK-APO was monodispersed in solution with a well-defined spherical morphology.

The VCR-loaded GKRK-APO stability in physiological conditions is a prerequisite for further application *in vivo*; therefore, 10% FBS in PBS was employed to mimic the *in vivo* conditions. The stability of the VCR-loaded GKRK-APO in the 10% FBS was evaluated using Turbiscan Lab^®^ Expert (Formulaction SA, Toulouse, France). According to this evaluation (Celia et al., 2009), the transmission or back-scattering profiles (less than 0.5%) obtained (Figure 1(G)) indicated there was no apparent aggregation or sedimentation of VCR-loaded GKRK-APO in the culture medium over 72 h. In addition, the above prepared nanocages could be stable in full rat serum (simulated physiological condition) for about 48 h.

### Optimization of peptide density

As the density of GKRK peptide in APO was a key factor that will influence the targeting efficiency of GKRK-APO greatly, the cellular uptake of Cy5.5-labled APO with modifications of different densities of GKRK peptide were evaluated in U87MG cells (over-expressed HSPG and TfR1) to guide the formulation optimizing process. As shown in Figure S1, when the peptide/APO molar ratio was 0.5%, there was no different between the cellular uptake of GKRK-APO and parent APO (*p* > .05). While the cellular uptake of APO was significantly influenced by the increase of peptide/APO molar ratio from 0.5% to 3%. With the further increase of the ratio to 6%, there was no significant difference in uptake compared with the APO with a 3% ratio. This was possibly caused by the saturation phenomenon of HSPG on cells. Limited by the number of receptors and the recycling of endocytosis, receptor-mediated endocytosis is a saturated pathway, which restricts the amount of the GKRK-APO that are available for cellular uptake. Considering the above results, the molar ratio of 3% for GKRK was selected in next experiments.

### *In vitro* cellular uptake and cytotoxicity assay

**Figure 2.**
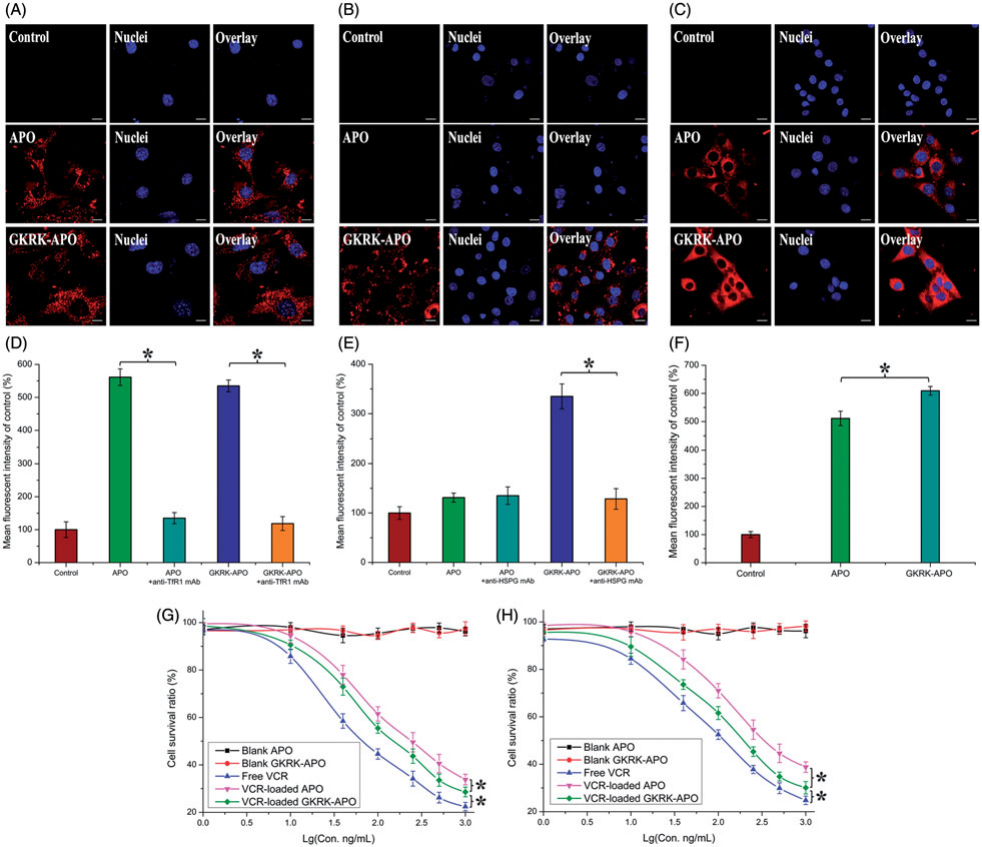
Cellular uptake of Cy5.5-labeled APO or Cy5.5-labeled GKRK-APO by bEND.3 cells (A and B), HUVECs (C and D), and U87MG cells (E and F). Cy5.5-positive cells were calculated by a FCM, and intracellular fluorescence was captured by a CLSM. Scale bars represent 10 μm. The cytotoxicity of U87MG cells (G) and HUVEC cells (H) cultured with various samples. The data are presented as the means ± SD (n = 3). * indicates P< 0.05. Scale bars represent 10 μm.

Cellular uptake of Cy5.5-labled GKRK-APO or Cy5.5-labled APO by bEnd.3, HUVEC, and U87MG cells was conducted to assess the targeting property. bEnd.3 cells, the main component of the BBB (Hu et al., 2009), were selected as the TfR1-positive cell type used to investigate the BBB targeting ability of nanocarriers. As shown in Figure 2(A,B), the very similar fluorescence intensities was observed in bEnd.3 cells incubated with GKRK-APO and APO. In addition, the binding was significantly inhibited by an anti-TfR1 mAb (Millipore, Burlington, MD), indicating specific binding of parent APO to TfR1 on bEnd.3 cells. HUVEC cells were used as the model cells of tumor angiogenesis to confirm the neovasculature targeting ability of GKRK peptide. As expected, GKRK peptide enhanced the uptake of GKRK-APO by HUVEC cells (Figure 2(C,D)) based on the results of CLSM and FCM. Moreover, the uptake efficiency of APO was not ideal. The mean fluorescence intensity of the formulation declined to a level similar to that of control. By contrast, the binding was significantly inhibited by excess anti-HSPG mAb (Millipore) and the intracellular fluorescence of GKRK-APO declined to a level similar to that of APO. The results demonstrated that when the expression level of HSPG on the cell surface was lower, GKRK-APO could not efficiently recognize and bind with the target cell via the GKRK motif, and thus, the uptake efficiency of GKRK-APO was not ideal. To evaluate the glioma-targeting efficiency, U87MG cells were used to measure the cellular uptake of nanocarriers. As shown in Figure 2(E,F), APO displayed significant internalization into U87MG cells, while even higher accumulation was observed in cells incubated with GKRK-APO. The results suggested that glioma cell recognition using dual mediations (TfR1 and HSPG-mediated endocytosis) was enhanced compared with that of the parent APO (TfR1-mediated endocytosis). The results could be a consequence of the synergism between TfR1 and HSPG mediations and were consistent with the data from the FCM analysis.

The cytotoxicities of blank APO, blank GKRK-APO, and different VCR formulations were evaluated with the MTT assay after incubation with HUVEC cells and U87MG cells for 72 h. As shown in Figure 2(G,H), the blank GKRK-APO and blank APO had very little toxic effects against the aforementioned cells, which revealed that the GKRK-APO and APO were relatively safe. This result also demonstrated that GKRK peptide had no effect on cell viability. With increased concentrations of VCR, free VCR could result in obvious antiproliferative effects to HUVEC cells and U87MG cells; thus, proving the anticancer effect on such kind of brain tumors. In addition, the free VCR group displayed the greatest cytotoxicity in both U87MG cells and HUVEC cells (IC_50_ values of 16.74 ng/mL (Figure 2(G)) and 22.83 ng/mL (Figure 2(H)), respectively). It could be concluded that free VCR could be quickly transported into cells by passive diffusion with a high concentration gradient (10–1000 ng/mL) under *in vitro* conditions. However, VCR-loaded GKRK-APO and VCR-loaded APO underwent possible lysosome-based drug release process after entering the intracellular region. Therefore, free VCR exhibited a stronger inhibitory effect on the proliferation of monolayer U87MG cells and HUVEC cells compared with VCR-loaded apoferritin-based nanocarriers. For VCR-loaded GKRK-APO and VCR-loaded APO, the improved cellular uptake led to an anticipated enhanced cytotoxicity effect. This showed that the delivery of VCR by the GKRK-APO significantly increased the cytotoxicity (IC_50_ values of 25.21 ng/mL and 28.14 ng/mL on U87MG cells and HUVEC cells, respectively) when compared with APO (33.65 ng/mL and 42.28 ng/mL on U87MG cells and HUVEC cells, respectively). The cytotoxicity studies indicated that the synergistic effect of GKRK peptide and parent APO promoted anti-proliferative activities in U87MG cells that over-expressed TfR1 and HSPG. These results from the cytotoxicities of VCR-loaded GKRK-APO and VCR-loaded APO were consistent with the *in vitro* cellular uptake results shown in Figure 2(C–F).

As we know, the singular properties of proteins are related to their specific structures. The surface modifications of apoferritin in this paper may alter its conformation and affect its activity. As a concept verifying experiment, we did not survey the conformation changes of apoferritin after modifying by GKRK peptide. However, the results of our paper revealed that GKRK-APO had similar drug loading and releasing profiles, TfR1 targeting ability, and cytotoxicity as APO did. For this, the GKRK modified apoferritin did not change its activities that we desired. More studies are needed to explore this problem in the future.

### Penetration across the *in vitro* BBB and BBTB models

**Figure 3.**
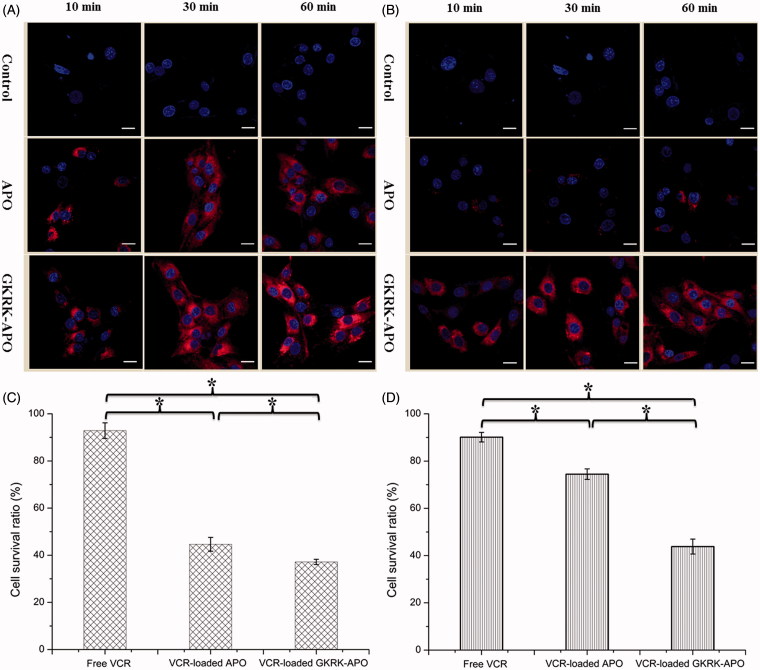
Uptake of APO or GKRK-APO on the in vitro co-culture model. CLSM images of U87MG cells uptake with Cy5.5-labed APO or Cy5.5-labed GKRK-APO after crossing the co-culture BBB model (A) and co-culture BBTB model (B). The cytotoxicity of free VCR, VCR-loaded APO and VCR-loaded GKRK-APO after crossing the co-culture BBB model (C) and co-culture BBTB model (D). The data are presented as the means ± SD (n = 3). * indicates P< 0.05. Scale bars represent 10 μm.

The BBB is a major physiological barrier that prevents drugs or drug delivery systems from entering the brain targeted region. Therefore, an *in vitro* co-culture model of bEnd.3/U87MG cells was constructed to estimate the penetration efficiency of various samples in mimicking conditions *in vivo*. Cellular uptake of Cy5.5-labeled GKRK-APO or Cy5.5-labeled APO in U87MG cells was investigated by CLSM. As shown in Figure 3(A), the fluorescence signals of Cy5.5 in GKRK-APO or APO were observed at each point, indicating that APO could cross the BBB and promote uptake in U87MG cells. Another barrier is the BBTB, which restricts accumulation of drugs or drug delivery systems in glioma, exacerbating the failure of chemotherapy. To better imitate the BBTB, an HUVEC/U87MG cells co-culture model was set up to explore the targeting ability and transcytosis efficiency of various samples. As shown in Figure 3(B), the fluorescence signals of Cy5.5 in GKRK-APO were significantly higher than that of APO at all predetermined time points. The present results suggested that APO with GKRK peptide modification possessed the targeting ability to the BBB and BBTB.

Consistent with these results, cytotoxicity studies also confirmed the significant penetrating effect of APO on cellular uptake in the BBB and BBTB co-culture model. As shown Figure 3(C), after addition of the free VCR, VCR-loaded GKRK-APO, and VCR-loaded APO, the survival (%) of U87MG cells after crossing the bEnd.3 cells was 92.86 ± 3.33%, 44.64 ± 2.94%, and 37.15 ± 1.12%, respectively. The results indicated that both GKRK-APO and APO possessed the targeting ability to the U87MG cells after crossing the BBB model via TfR1. While, the free VCR did not cross the BBB model at all. In the BBTB model (Figure 3(D)), as expected, VCR-loaded GKRK-APO significantly increased the cytotoxicity of U87MG cells after crossing the HUVEC cells, with a survival (%) of 43.82 ± 3.17% compared to 90.11 ± 2.02% and 74.47 ± 2.23% for free VCR and APO, respectively. These results suggested that GKRK-APO exhibited a significant inhibitory effect by transporting drug across HUVEC cells and then targeting U87MG cells. Although free VCR could inhibit the growth of monolayer U87MG cells and HUVEC cells more strongly than VCR-loaded apoferritin-based nanocarriers in the *in vitro* cytotoxicity assay (Figure 2(G,H)), VCR-loaded GKRK-APO could more strongly inhibit the growth of U87MG cells after crossing BBB model and BBTB model than free VCR in these models, which could be attributed to GKRK selectivity and parent APO delivery.

### Pharmacokinetic and tissue distribution studies

The plasma concentration-time profiles of VCR after i.v. administration of free VCR and VCR-loaded nanocarriers was shown in Figure S2(A). It was found that VCR-loaded GKRK-APO and VCR-loaded APO showed similar plasma concentration-time profiles. Both the VCR-loaded GKRK-APO and VCR-loaded APO showed initial high blood circulating levels (0.08 h after injection), while free VCR was quickly cleared from the systemic circulation. The pharmacokinetic parameters are listed in Figure S2(B). VCR-loaded GKRK-APO demonstrated significantly slower clearance rate (CL) and higher AUC when compared with free VCR. Furthermore, there was no remarkable different in CL and AUC between the VCR-loaded GKRK-APO and VCR-loaded APO (*p* > .05). These results indicate that the conjugation of an adequate amount of GKRK peptide on the surface of APO did not impair the long-circulation characteristic of parent APO.

The selective distribution of drug-loaded nanocarriers in tumors could potentially benefit the antitumor activity of chemotherapy *in vivo*. To verify this, tissue distribution was evaluated in nude mice with intracranial U87MG glioma following i.v. administration of free VCR, VCR-loaded APO, and VCR-loaded GKRK-APO, respectively. As shown in Figure S2(C), the concentrations of VCR in the brain of VCR-loaded GKRK-APO was 6.5-fold over that of free VCR, and 1.4-fold over that of VCR-loaded APO, respectively. As shown in Figure S2(D), VCR-loaded GKRK-APO exhibited a similar tissue distribution profile with the VCR-loaded APO in the non-targeted tissue, while a significantly higher level of free VCR was observed accumulated in the liver, heart, spleen, and kidney. Due to the existence of BBB, the levels of VCR in brain are very low, compared with all the other tissues (Figure S2(C) vs. D: 0.05–0.3% vs. 2.3–6.4%). The circulating time of VCR encapsulated in apoferritin nanocage in blood was prolonged. Hence, it indicated that the body distribution of VCR-loaded APO mainly depended on the distribution behavior of the parent apoferritin *in vivo*. What is different from VCR-loaded APO was that VCR-loaded GKRK-APO modified with GKRK peptide had significantly enhanced glioma targeting delivery and resulted in higher drug concentration in brain. These results from mice were consistent with the *in vitro* results shown in Figure 3. The GKRK-APO could be used for further study in the treatment of brain cancer *in vivo.*

### *In vivo* imaging and glioma distribution

**Figure 4.**
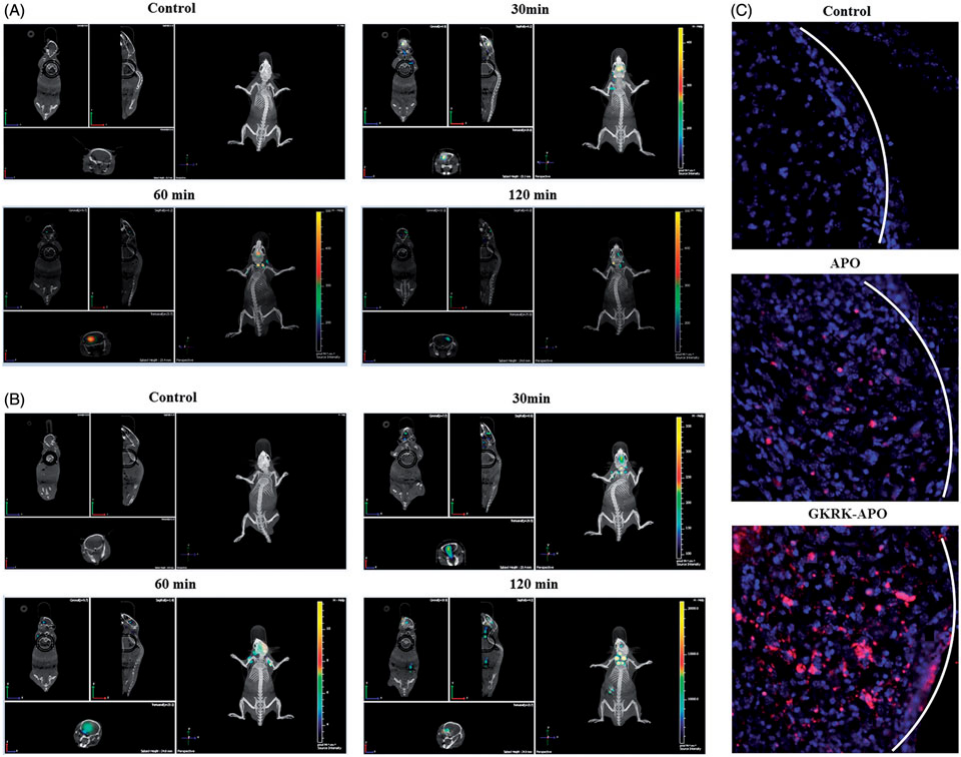
Biodistribution of Cy5.5-labeled GKRK-APO (A) and Cy5.5-labeled APO (B) in mice bearing intracranial U87MG glioma determined by an IVIS^®^ Spectrum-CT. Distribution of Cy5.5 in the brain of mice bearing intracranial U87 glioma determined by a CLSM (C). The white line shows the margin of intracranial glioma. The red represents Cy5.5 and the nuclei were stained by DAPI (blue).

**Figure 5.**
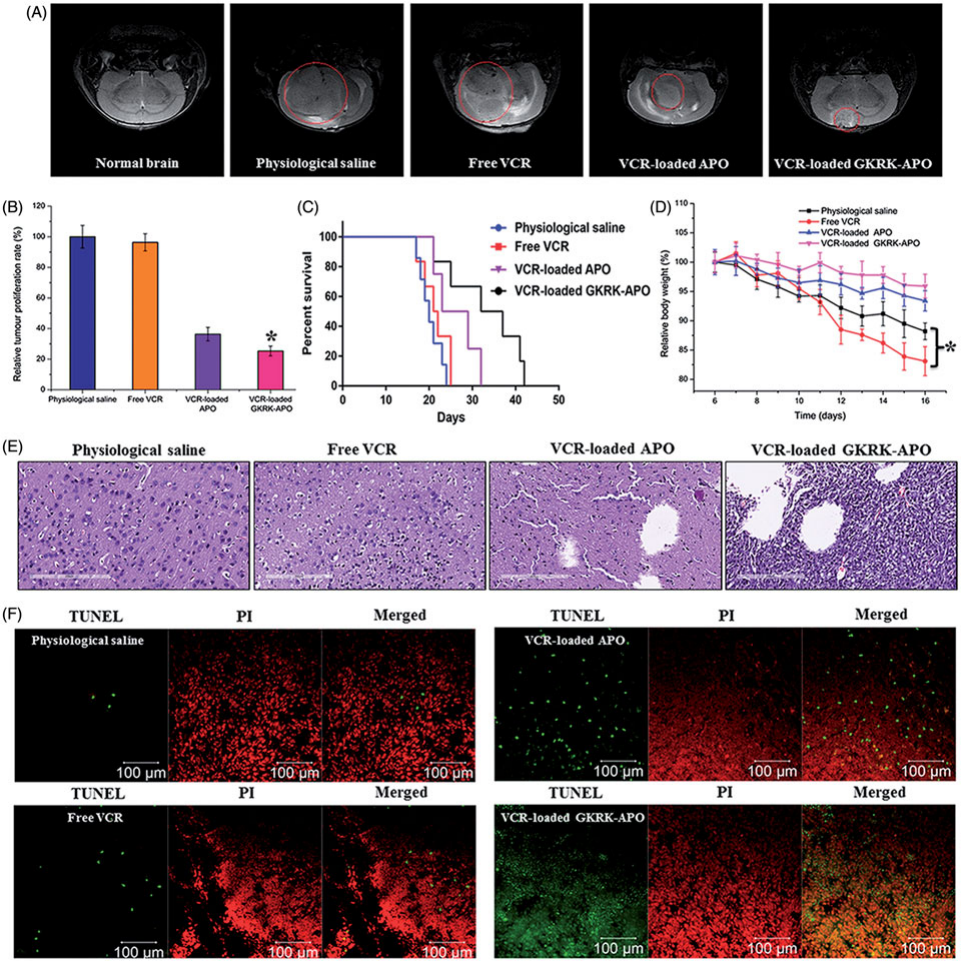
Anticancer efficacy in intracranial U87MG glioma-bearing mice. Notes: Efficacy after treatment with various formulations with a dose of 1 mg/kg VCR at days 8, 10, 12, and 14 from inoculation. MRI of normal and pathological brains at 16 day after inoculation (A). Inhibition of the brain glioma volume (B). Kaplan- Meier survival curves (C). Body weight changes (D). HE staining (E) and TUNEL analysis (F) of brain tumours. Red (the colour spots in PI): cell nuclei. Green (the colour spots in TUNEL): apoptosis cells. The data are presented as the means ± SD (n =6). * indicates P< 0.05.

The brain targeting efficiency of GKRK-APO was investigated in nude mice with intracranial U87MG glioma. After the Cy5.5-labeled nanocarriers were given through the tail vein, time-dependent biodistribution of the GKRK-APO and APO were observed using non-invasive NIR fluorescence imaging in live animals. Based on *in vivo* imaging, the brain accumulation was much higher for the GKRK-APO (Figure 4(A)) and APO (Figure 4(B)) group. While, there were no signals in the brain of control group (as the brains in control group showed no signals at the time points of 30, 60, and 120 min, only one picture was selected to present the whole results). These initial data provided substantial evidence that apoferritin nanocage (GKRK-APO and APO) efficiently crossed the BBB and exhibited good brain targeting ability *in vivo* (the tumor induced in brain please see in Figure 5(A)). In addition, GKRK-APO showed a relatively higher accumulation in the brain. The trend observed for *in vivo* imaging in mice was consistent with the results of the tissue distribution studies (Figure S2(C)).

To further evaluate its *in vivo* glioma targeting capability, immunofluorescence assay was conducted after the treatment of various Cy5.5-labeled samples in mice bearing intracranial glioma. As shown in Figure 4(C), in the APO group, fluorescent intensity was distributed in the brain, suggestive of APO across the BBB. GKRK-APO showed a slightly higher accumulation than APO in the glioma region, indicating the precise glioma targeting property of GKRK-APO with the modification of ligands. These results are consistent with the *in vivo* imaging results (Figure 4(A,B)) and indeed support our hypothesis that the GKRK-APO could not only cross the BBB but also cross the BBTB and selectively target the glioma cells. The results again emphasized the advantage of the GKRK-APO in glioma targeting delivery.

### *In vivo* therapeutic efficacy

The *in vivo* antiglioma efficacy was investigated using the mice bearing intracranial U87MG glioma. After treatment with the control formulations (physiological saline, free VCR, and VCR-loaded APO) and VCR-loaded GKRK-APO, overall antiglioma efficacy was observed by MRI for monitoring cancer volume and was confirmed using survival curves. Consistent with the results of glioma distribution (Figure 4(C)), tumor inhibition analysis confirmed the significant glioma-targeting effect of VCR-loaded GKRK-APO in mice with intracranial U87MG glioma. As shown in Figure 5(A), glioma diameter in the brain at day-16 was clearly reduced according to MRI after treatment with the VCR-loaded GKRK-APO as compared with those after treatment with control formulations, suggestive of VCR-loaded GKRK-APO across the multiple physiological barriers and targeting glioma cells. Relative tumor proliferation rate at day 16 (Figure 5(B)) was 100.00 ± 7.38% for physiological saline, 96.34 ± 5.56% for free VCR (because insufficient drug does reach to the tumor), 36.31 ± 5.52% for VCR-loaded APO, and 25.33 ± 6.52% for VCR-loaded GKRK-APO. These results indicate that the therapeutic efficacy of the VCR-loaded GKRK-APO is significantly superior to that of other formulations in intracranial U87MG glioma-bearing mice models.

The clinical therapeutic benefits are mainly determined based on the quality of life and prolonged survival time of cancer patients. In further investigation of the potential of GKRK-APO in antiglioma therapy *in vivo*, the Kaplan–Meier survival curve of intracranial U87MG glioma-bearing mice was used (Figure 5(C)). As expected, treatment with VCR-loaded GKRK-APO significantly prolonged the median survival time (35 days), which was 1.8, 1.6, and 1.3-fold higher than that of physiological saline, free VCR, and VCR-loaded APO, respectively (*p* < .05). This was mainly attributed to the target systemic delivery of GKRK-APO, which was demonstrated by *in vivo* imaging (Figure 4(A–C)). In addition, the body weight variation of mice was monitored during the experimental period (Figure 5(D)). More than 15% of weight loss was found in the free VCR group at the end of experimental period. The weight loss of these free drug groups were likely due to the non-targeted characteristics and tumor cachexia. While, the much smaller weight loss of mice in VCR-loaded GKRK-APO group than that of free VCR group during the whole experimental period, indicated the GKRK peptide modified apoferritin nanocage reducing unspecific cellular uptake through glioma-targeted delivery.

Histological changes of glioma after different treatments were detected and compared using HE staining. As shown in Figure 5(E), glioma from mice treated with VCR-loaded GKRK-APO displayed abnormal tissue and cells, exhibiting a hypocellular and necrotic zone, whereas tumors from mice treated with the other formulations showed a more hypercellular zone and normal nuclear polymorphism. Cell apoptosis of glioma after different treatment was also detected by TUNEL assay. As shown in Figure 5(F), although ignorable necrotic foci were elicited by the physiological saline group, the number of apoptotic cells (green) increased after treatment with VCR-containing formulations. Compared with treatment of free VCR or VCR-loaded APO, treatment with the VCR-loaded GKRK-APO induced the widest zone of cell apoptosis, showing the most effective activity in inducing tumor apoptosis. Overall, these results indicated that the GKRK-APO had a more effective antiglioma activity. The trend observed for the histological analysis was consistent with the results of the biodistribution (Figure 4(A–C)) and the antiglioma efficacy *in vivo* (Figure 5(A–C)).

### Safety assay

The goal for a nanocarrier is to achieve optimal therapeutic efficacy with acceptable safety profiles during *in vivo* applications. To evaluate the toxicity of VCR-loaded GKRK-APO, the healthy mice were treated with PBS, free VCR, VCR-loaded GKRK-APO, and VCR-loaded APO. The representative organs including brain, heart, liver, kidney, and lung were collected and stained with H&E for histology study. As shown in Figure S3, no indicators of damage were observed for these organs after treatments, suggesting that all treatments did not cause systemic toxicity. Considering therapeutic efficacy, GKRK-APO is a safe and an effective drug delivery system for glioma therapy.

## Conclusions

In this study, we designed natural nanoscale drug delivery platform (GKRK-APO) achieving systemic glioma-targeted drug delivery by employing GKRK peptide. GKRK-APO could penetrate BBB and BBTB and target glioma cells. VCR-loaded GKRK-APO could effectively enhance the antiglioma efficacy *in vitro* and *in vivo*. In recent years, apoferritin has been used as nanoplatforms for tumor-targeted diagnosis and therapy by many groups. However, most studies have focused on peripheral tumors rather than brain tumors, and to the best of our knowledge, this is the first time the combination of the dual brain glioma-targeting delivery by a specific ligand and parent apoferritin has been reported. Although the current strategy exhibits an effective glioma-targeting delivery and antiglioma effect, there remain some shortcomings for the delivery system. For example, the presence of ligand could interfere with the self-assembling process of apoferritin, resulting in a low yield of the final products. Thus, the surface engineering technique for apoferritin needs to be improved. In a future study, we will continue to perform *in vitro* and *in vivo* evaluations and further explore the application of GKRK-APO in glioma-targeted delivery.

## Supplementary Material

IDRD_YANG_et_al_Supplemental_Content.doc
