# Supplementary material for: Glioma targeting peptide modified apoferritin nanocage
Source: Drug Deliv. 2018 May 4;25(1):1013–24. doi: 10.1080/10717544.2018.1464082 (PMC6058491; doi:10.1080/10717544.2018.1464082)
Supplement: IDRD_YANG_et_al_Supplemental_Content.doc [file IDRD_A_1464082_SM9504.doc]

- **Supplementary information**

**
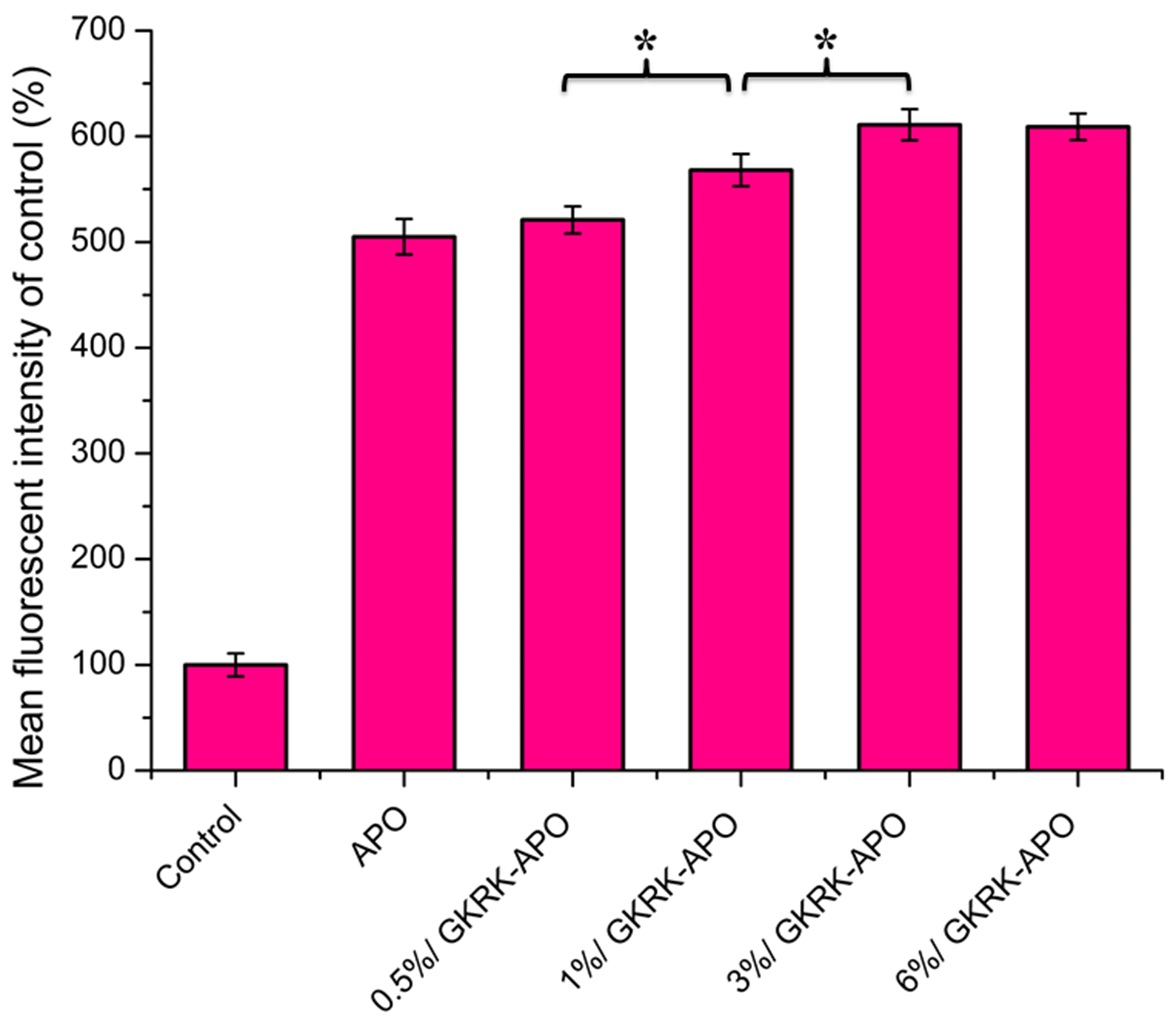
**

**Figure S 1.** Cellular uptake of Cy5.5-labeled GKRK-APO with different densities of GKRK peptides in U87MG cells after incubation for 2 h at 37 °C. The autofluorescence of the cells was applied as the control. The data are presented as the means ± SD (n = 3). * indicates P< 0.05.

**
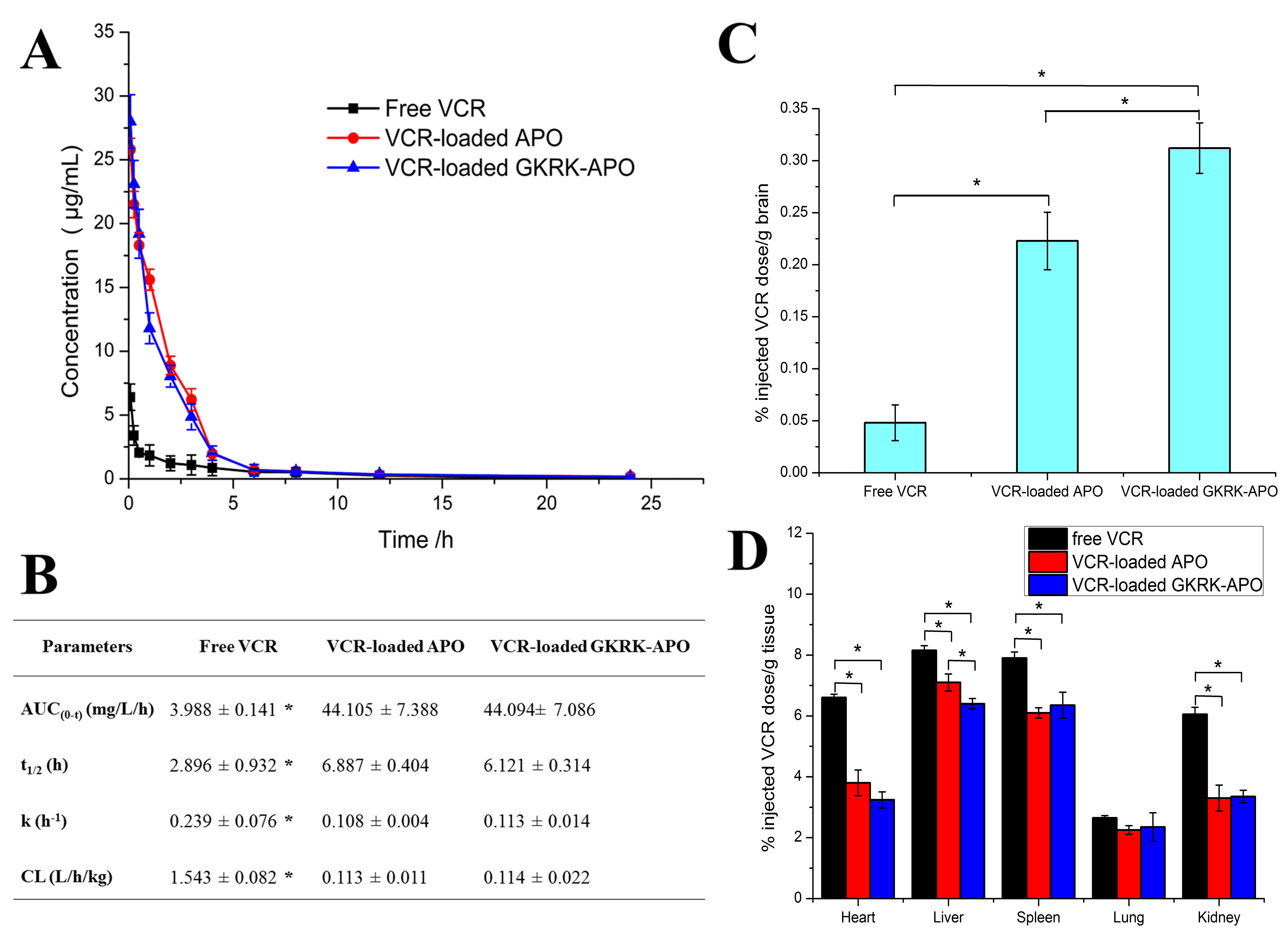
**

**Figure S 2**. Plasma VCR concentration-time profiles (A) and pharmacokinetic parameters (B) after i.v. injection of different formulations in rat (n=3). The distribution of VCR in brain 0.5 h after i.v. injection (C). Concentration of VCR in organs 0.5 h after i.v. injection (D). The data are presented as the means ± SD (n = 3). * indicates P< 0.05.

**
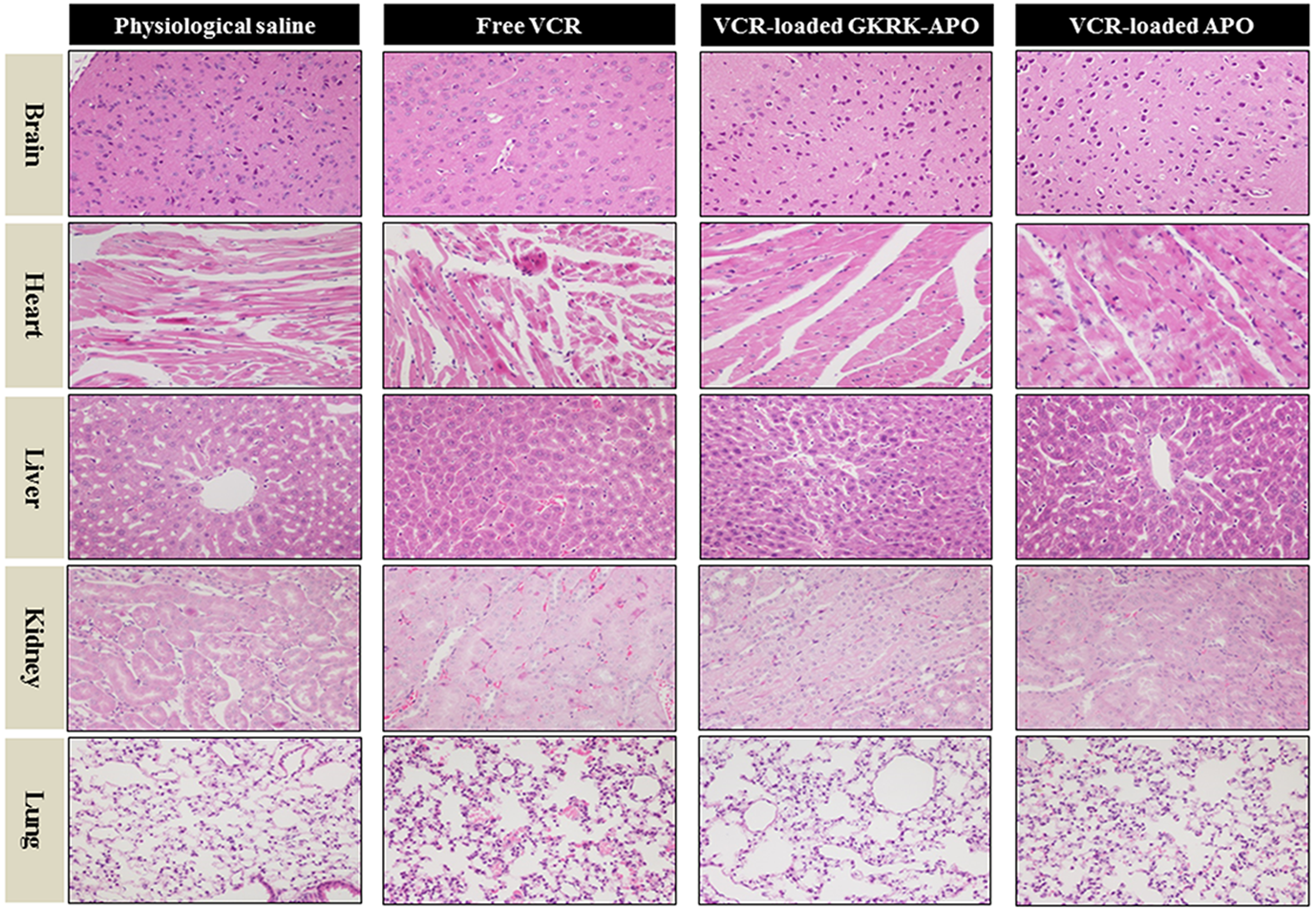
**

**Figure S 3**. Histological staining of organs from mice treated with different formulations.
